# The Development of Translational Biomarkers as a Tool for Improving the Understanding, Diagnosis and Treatment of Chronic Neuropathic Pain

**DOI:** 10.1007/s12035-017-0492-8

**Published:** 2017-03-30

**Authors:** David A. Buckley, Elaine M. Jennings, Nikita N. Burke, Michelle Roche, Veronica McInerney, Jonathan D. Wren, David P. Finn, Patrick C. McHugh

**Affiliations:** 1 Centre for Biomarker Research and Department of Pharmacy, School of Applied Sciences, University of Huddersfield, Huddersfield, HD1 3DH UK; 2 Pharmacology and Therapeutics, National University of Ireland, Galway, Ireland; 3 Centre for Pain Research and Galway Neuroscience Centre, National University of Ireland, Galway, Ireland; 4 Physiology, School of Medicine, National University of Ireland, Galway, Ireland; 5 University Hospital Galway, Galway, Ireland; 6 Arthritis and Clinical Immunology Research Program, Oklahoma Medical Research Foundation, Oklahoma City, OK USA

**Keywords:** Neuropathic pain, Biomarker, Plasma, Dorsal horn, Back pain, Inflammatory pain

## Abstract

Chronic neuropathic pain (CNP) is one of the most significant unmet clinical needs in modern medicine. Alongside the lack of effective treatments, there is a great deficit in the availability of objective diagnostic methods to reliably facilitate an accurate diagnosis. We therefore aimed to determine the feasibility of a simple diagnostic test by analysing differentially expressed genes in the blood of patients diagnosed with CNP of the lower back and compared to healthy human controls. Refinement of microarray expression data was performed using correlation analysis with 3900 human 2-colour microarray experiments. Selected genes were analysed in the dorsal horn of Sprague–Dawley rats after L5 spinal nerve ligation (SNL), using qRT-PCR and ddPCR, to determine possible associations with pathophysiological mechanisms underpinning CNP and whether they represent translational biomarkers of CNP. We found that of the 15 potential biomarkers identified, tissue inhibitor of matrix metalloproteinase-1 (*TIMP1*) gene expression was upregulated in chronic neuropathic lower back pain (CNBP) (*p* = 0.0049) which positively correlated (*R* = 0.68, *p* = ≤0.05) with increased plasma TIMP1 levels in this group (*p* = 0.0433). Moreover, plasma TIMP1 was also significantly upregulated in CNBP than chronic inflammatory lower back pain (*p* = 0.0272). In the SNL model, upregulation of the *Timp1* gene was also observed (*p* = 0.0058) alongside a strong trend for the upregulation of melanocortin 1 receptor (*p* = 0.0847). Our data therefore highlights several genes that warrant further investigation, and of these, *TIMP1* shows the greatest potential as an accessible and translational CNP biomarker.

**Electronic supplementary material:**

The online version of this article (doi:10.1007/s12035-017-0492-8) contains supplementary material, which is available to authorized users.

## Introduction

Chronic neuropathic pain (CNP) is a physically debilitating and pathologically complex disorder featuring maladaptive cellular responses and the subsequent development of ectopic discharge and neuronal hyperexcitability [1]. This manifests as allodynia and hyperalgesia in up to 50% of patients with CNP [2]. CNP is often a consequence of traumatic nerve injury but is also associated with numerous peripheral and centrally mediated factors, such as diabetes, multiple sclerosis and stroke [3, 4], and results in a complex plethora of CNP subtypes including chronic neuropathic lower back pain. Therefore, CNP diagnosis is inherently complex and remains subjective, with the primary methods for diagnosis being the painDETECT [5], Neuropathic Pain Questionnaire (NPQ) [6] and the Leeds Assessment of Neuropathic Symptoms and Signs (LANSS) screening tools [7], alongside clinical assessment. The stark deficit in effective treatments and objective diagnostic tools therefore provides the impetus to determine novel and translational diagnostic biomarkers to promote early intervention in CNP.

There is a growing body of evidence which suggests an increasing involvement of non-neuronal mechanisms in the pathophysiology of CNP. These often pertain to the immune system to such a degree that CNP has been considered a neuro-immune disorder with glial cell and cytokine involvement [8, 9]. The diversity, complexity and involvement of these non-neuronal mechanisms associated with CNP may therefore herald the opportunity to determine an accessible biomarker in blood. Much of the research searching for CNP biomarkers in humans to date has considered cerebrospinal fluid [10] and the brain [11] with functional magnetic resonance imaging (fMRI) being proposed as a way of determining CNS biomarkers to guide clinical practice [12]. However, such methodology is likely to be impractical as acceptable and cost-effective method of biomarker detection in clinical practice.

The majority of gene expression studies have focused solely on animal models of neuropathic pain (NP) [13], ranging from chronic constriction injury (CCI) [14] and spinal nerve ligation (SNL) [15] to the spared nerve injury [16] and drug-induced neuropathy [17] models. Such studies have identified extensive groups of genes whose expression is altered after nerve injury [18] and have provided valuable insights into the mechanisms underpinning the development and maintenance of CNP. Little though has been translated to advances in diagnostic biomarkers in human CNP subjects. A pertinent previous study sought to elucidate biomarkers by determining correlations in gene expression changes between rat blood and ipsilateral lumbar dorsal quadrant (iLDQ) after CCI, using bioinformatics in conjunction with microarray analysis, which demonstrated the potential of blood transcriptomic changes as peripheral markers to reflect those in the iDLQ after nerve injury [19].

In order to bridge this gap to humans, we sought to determine the feasibility of a minimally invasive method of sample collection by performing microarray analysis to establish a panel of genes differentially expressed in blood from a cohort of patients diagnosed with CNP of the lower back and healthy human controls. Due to the complexities of CNP, we focused our study on the chronic lower back pain (CLBP) of neuropathic origin. CLBP is a common chronic pain condition and has been estimated to account for the biggest proportion of the chronic pain market [20]. Moreover, ∼85% of CLBP patients are grouped as non-specific CLBP [21], and at present, there is a lack of objective diagnostic tools to determining if this non-specific CLBP group is of a neuropathic or inflammatory origin. Thus, there is a clear need to identify biomarkers that can help delineate these subtypes that could also potentially provide insight into other CNP subtypes and pain conditions. After our initial array analysis in human samples, we investigated the expression of a subset of the candidate genes identified in the dorsal horn of sham and L5 SNL Sprague–Dawley rats. This strategy therefore prioritised the search for viable biomarkers in human blood whilst determining if these biomarkers are translational across species. The L5 SNL Sprague–Dawley rat model was chosen as the translational model of CNP as symptoms generated in in this system mimic the neuropathic pain symptoms of human patients [15, 17]. Furthermore, we focused on the dorsal horn region of the spinal cord as these neurons process sensory information and undergo changes that contribute to the development and maintenance of neuropathic pain [22]. Using the translational approach to biomarker discovery also highlights whether these molecules may be perturbed in the mechanisms underpinning CNP and therefore whether they may hold potential as disease phenotypic indicators and/or pharmacological targets in preclinical analgesic drug development.

## Materials and Methods

### Human Clinical Samples

PAXgene Blood RNA Tubes (IVD; PreAnalytiX GmbH, Hombrechtikon, Switzerland) from 10 individuals with chronic neuropathic lower back pain (CNBP) lasting for more than 6 months were obtained through ProteoGenex tissue procurement services (Culver City, CA), alongside a further 10 age-gender matched controls also acquired through ProteoGenex tissue procurement services. Patients were recruited after clinical assessment of their pain symptoms, including CT scans, MRI scans and electroneuromyography. Pain intensities were determined using the verbal rating scale (VRS) [23]. All patients were non-responsive to non-narcotic and anti-inflammatory analgesics. Plasma was obtained using BD Vacutainer K2-EDTA tubes with centrifugation at 1000×*g* for 10 min and immediate storage of the plasma at −80 °C. Patients with major psychiatric disorders, cancer or diabetes were excluded from this study. Donor consent was obtained through ProteoGenex under Protocol PG-ONG2003/1, titled Collection of Tissue, Blood and Bone Marrow. Plasma from a total of 12 patients with chronic inflammatory back pain (CIBP) was also obtained to delineate between a potential translational biomarker of CNBP and one of CIBP. The absence of CNBP was determined by consultant assessment and the post-consultation completion of the S-LANSS questionnaire, which allows delineation between nociceptive and neuropathic pain [24] and shows good correlation with other screening tools for lower back-related pain [25]. Patients with an S-LANSS score of 12 or greater were excluded. Pain severity was determined using the Chronic Pain Grade questionnaire [26] and is presenting with demographic data on the patient and control groups (Supplementary Table 1).

### RNA Isolation

Total RNA was isolated from the PAXgene Blood RNA Tubes using the Preserved Blood RNA Purification Kit II (Norgen, Biotek, ON, Canada) according to the manufacturer’s instructions. In brief, the RNA was treated with DNAse and purified on columns. RNA concentration was measured on a NanoDrop ND2000 ultraviolet–visible spectrophotometer (Labtech International Ltd., UK), and RNA integrity was checked on an Agilent 2100 Bioanalyzer (Agilent Technologies, Amsterdam, The Netherlands). RNA was judged as suitable for gene expression analysis only if samples showed intact bands of 28S and 18S ribosomal RNA subunits, displayed no chromosomal peaks or RNA degradation products and had an RNA integrity number (RIN) above 7.0.

### Affymetrix Microarray and Data Analysis

Total RNA was labeled using an Ambion WT Expression kit (Life Technologies, Bleiswijk, The Netherlands) and hybridised to Affymetrix Human Gene 1.0 ST expression arrays (Affymetrix, Santa Clara, CA, USA). Sample labeling, hybridization to chips and image scanning were performed according to the manufacturer’s instructions on an Affymetrix GeneTitan instrument. Quality control was performed using Affymetrix Expression Console, and interpretation of data was facilitated by Affymetrix Transcriptome Analysis Console 2.0 (TAC2.0). Transcripts exhibiting a fold change of ≥1.2 and a *p* value of ≤0.05 (ANOVA) were considered differentially expressed and suitable for further correlation analysis and refinement.

### Analysis of General Gene–Gene Correlations

A total of 3900 human 2-colour microarray experiments were downloaded from NCBI’s Gene Expression Omnibus (GEO) and normalised as described previously [27]. The experiments span different tissues and different conditions—these 3900 were chosen because it is the subset of all 2-colour arrays that have been curated by NCBI staff. Two-colour arrays were chosen because they reflect how gene expression differs between two conditions, usually experimental and control, which emphasises how genes are correlated in their response. Gene–gene Pearson’s correlation coefficients were calculated using only the experiments where the two genes were present on the same microarray.

### Further Refinement of Expression Data

In order to determine the genes with the greatest evidence for involvement in CNBP, refinement of genes was undertaken with specified criteria, which includes a greater statistical stringency (Table 1), the presence of a gene within our correlation analysis output, and finally, whether there is a body of literature pertaining to the role of the molecule in pain pathways. Literature was searched to include all publications available up to, and including, February 2017, using both PubMed and general electronic information databases with the gene name or symbol, along with the terms ‘pain’, ‘neuropathic’ or ‘neuropathic pain’.

**Table 1:** CNP biomarker panel of transcripts differentially regulated in human whole blood^a^

Array ID	Accession number	Gene name	Gene symbol	*p* value	FC in CNP	CA	Literature
7951385	NM_004347	Caspase 5	*CASP5*	0.0449	↑2.23	No	[39, 40]
8149927	NM_001831	Clusterin	*CLU*	0.0489	↑1.85	No	[41, 42]
7941621	NM_005700	Dipeptidyl-Peptidase 3	*DPP3*	0.0028	↑1.50	No	[37, 38, 43, 44]
7908793	NM_004433	E74-Like Factor 3	*ELF3*	0.0095	↑1.62	No	[45, 46]
7937707	NR_026643	Family with sequence similarity 99, member A	*FAM99A*	0.0017	↑1.64	No	–
8070720	NM_015259	Inducible T cell co-stimulator ligand	*ICOSLG*	0.0007	↑1.20	No	[19]
8065011	NM_024674	Lin-28 homolog A (*C. elegans*)	*LIN28A*	0.0183	↓1.50	No	[47]
7998055	NM_002386	Melanocortin 1 Receptor	*MC1R*	0.0005	↑1.40	No	[48–53]
8051396	NM_021209	NLR family CARD domain-containing protein 4	*NLRC4*	0.0437	↑1.99	No	[54]
8157450	NM_000608	Orosomucoid 2	*ORM2*	0.0225	↑1.97	Yes	–
7982287	NM_001039841	Rho GTPase activating protein 11B	*ARHGAP11B*	0.0025	↑1.57	No	–
8075477	NM_152267	Ring finger protein 185	*RNF185*	0.0032	↓1.68	No	–
7967972	NG_043316	RNA, U6 small nuclear 76, Pseudogene	*RNU6-76P*	0.0049	↓1.54	No	–
8167185	NM_003254	TIMP metalloproteinase Inhibitor 1	*TIMP1*	0.0049	↑1.50	Yes	[35, 36, 55–60]
7924499	NM_003268	Toll-like receptor 5	*TLR5*	0.0428	↑1.75	No	[61, 62]

^a^Genes documented here and subsequently analysed in the SNL model either exhibited a *p* value of ≤0.005 and a fold change (FC) of ≥1.5 or were present in our correlation analysis (CA)/literature search with a *p* value of 0.005–0.05 and a FC of ≥1.5 or a *p* value of ≤0.005 and a FC of 1.2–1.5

### Plasma TIMP1 Quantification

In order to clarify if circulating levels of tissue inhibitor of matrix metalloproteinase-1 (TIMP1) varied between patients with CNBP, CIBP and healthy controls, a total of 32 plasma samples were subject to a TIMP1 enzyme-linked immunosorbent assay (ELISA) (Invitrogen, UK). The assay was performed according to manufacturer’s instructions. In total, 10 μl of plasma was diluted to 200 μl prior to the procedure, each sample was analysed in duplicate and absorbance data was obtained using an Infinite F50 microplate reader (Tecan, UK). Absorbance data were converted into plasma TIMP1 levels using the standards provided, and the data were analysed using GraphPad Prism 6.0 using a Mann–Whitney test and Kruskal–Wallis test (*p* = ≤0.05 considered statistically significant).

### Animal Husbandry, L5 SNL Surgery and Tissue Harvest

Adult male Sprague–Dawley rats (*n* = 18) (matched at 7–8 weeks of age upon delivery and 250–350 g at time of experimentation; Harlan, UK) were housed singly, with food and water available ad libitum and maintained at constant temperature (21 ± 2 °C) under 12 h cycling of light–dark exposure (lights on at 07.00 h). The e xperimental procedures were approved by the Animal Care and Research Ethics Committee, National University of Ireland, Galway, and carried out under license from the Department of Health in the Republic of Ireland and in accordance with EU Directive 2010/63. One week following delivery and acclimatization to the animal unit, animals underwent surgery after allocation into either L5 SNL (*n* = 10) or sham (*n* = 8) groups. In brief, the rats were anaesthetised under isoflurane anaesthesia (3% induction, 1.5–2% maintenance in 0.5 L/min O_2_), and upon exposure of the left L5 spinal nerve, a ligature was applied. Sham rats were treated identically, aside from ligation of the L5 nerve. Animals were maintained until 35 days post-surgery at which point euthanasia was performed by decapitation and tissue was harvested from the spinal cord dorsal horn ipsilateral to the side of nerve injury, snap-frozen on dry ice and stored at −80 °C. RNA was extracted from tissue using the NucleoSpin® RNA kit (Machery–Nagel) with on-column DNase treatment followed by storage at −80 °C.

### Quantitative Real-Time PCR

A total of 20 ng of RNA from each dorsal horn sample was used for reverse transcription and subsequent amplification using the QuantiTect Whole Transcriptome Kit (Qiagen, UK). This was performed according to manufacturer’s instructions and included an 8-h incubation stage for high yield cDNA synthesis. After serial dilution of the amplification product, quantitative real-time polymerase chain reaction (qRT-PCR) was performed using a CFX96 instrument (Bio-Rad Laboratories, UK). Analysis of samples was performed in triplicate with each 12-μl reaction containing 6 μl of iTaq™ Universal SYBR® Green Supermix (Bio-Rad Laboratories), 300 nM of each forward and reverse primer (Supplementary Table 2) and 5 μl of diluted cDNA. Incubation consisted of polymerase activation and DNA denaturation at 95 °C for 2 min followed by 40 cycles of denaturation at 95 °C for 5 s with annealing and extension at 60 °C (unless otherwise stated in Supplementary Table 2) for 30 s followed by fluorescence detection. Upon completion of thermal cycling, melt-curve analysis was performed to confirm reaction specificity. Baseline subtraction and determination of the threshold cycle (Cq) were performed using Bio-Rad CFX Manager 3.1 (Bio-Rad Laboratories). Data was subsequently analysed with qbase + software (Biogazelle, Belgium) using an unpaired *t* test. Normalization of expression data was performed using both *Atp5b* and *Ubc*. Of 12 reference genes analysed with the rat geNorm kit (Primerdesign, UK), both were found to be comparably highly stable (M = 0.419, CV = 0.145).

### Digital Droplet PCR

A total of 20 ng of RNA was reverse transcribed using the Verso cDNA synthesis kit (Thermo Fisher Scientific, UK). This was performed according to manufacturer’s instructions. Random hexamers and anchored oligo-dT were both included at a ratio of 3:1 (*v*/v); 0.5 μl of RT Enhancer per 10 μl reaction was also included, followed by incubation at 42 °C for 60 min and 95 °C for 2 min. The cDNA was subsequently diluted to 100 μl. Further dilutions were performed for the reference gene *Atp5b* to avoid saturation of the digital droplet PCR (ddPCR) system. All reagents and equipment used for ddPCR were from Bio-Rad Laboratories. Each 20 μl PCR reaction consisted of 10 μl of QX200™ ddPCR™ Evagreen Supermix, 250 nM of forward and reverse primer, 5 μl of diluted cDNA and nuclease free water. This was loaded in to a DG8™ Cartridge with accompanying DG8™ Gasket and 70 μl of QX200™ Droplet Generation Oil for Evagreen for droplet generation using a QX200™ Droplet Generator. 96-well plates were then sealed using pierceable foil plate seals with a PX1™ PCR plate sealer. A T100™ Thermal Cycler was used with the following cycling conditions: enzyme activation for 5 min at 95 °C, followed by 40 cycles of denaturation at 95 °C for 30 s and annealing/extension at 60 °C for 1 min. Signal stabilisation was achieved by cooling to 4 °C for 5 min, heating to 90 °C for 5 min. A ramp rate of 2 °C per second was required for each step in the PCR. Data was then obtained using a QX200™ Droplet Reader with ddPCR™ Droplet Reader Oil and QuantaSoft™ Software, version 1.7. Normalisation of data was performed by dividing the total number of transcript copies per 20 μl reaction, by the geometric mean of *Rpl13a* and *Ubc*. Data was analysed using GraphPad Prism 6.0 using an unpaired *t* test (*p* = ≤0.05 considered statistically significant).

## Results

### Human Blood Transcriptome Analysis

In order to determine differentially regulated genes, we used gene microarray analysis to determine the expression of intracellular RNA from whole blood in patients with CNBP and in healthy controls. Our analysis highlighted a diverse range of genes that may be perturbed in the development or maintenance of CNBP, which in turn may function as potential biomarkers of CNBP. These include genes pertaining to immune function, inflammatory response and extracellular matrix turnover (Supplementary Table 3).

### Correlation Analysis

We also sought to evaluate how the differentially expressed genes in this study were correlated with each other in prior microarray experiments, by analysis of 3900 human 2-colour arrays obtained from NCBI’s GEO database. Global correlations (i.e. correlations not dependent upon the experimental condition being studied) between genes suggest their involvement in a common transcriptional response network. Similarly, negatively correlated gene sets may suggest how the response under the conditions being studied here (CNBP) differs from the general trends.

**Fig. 1:**
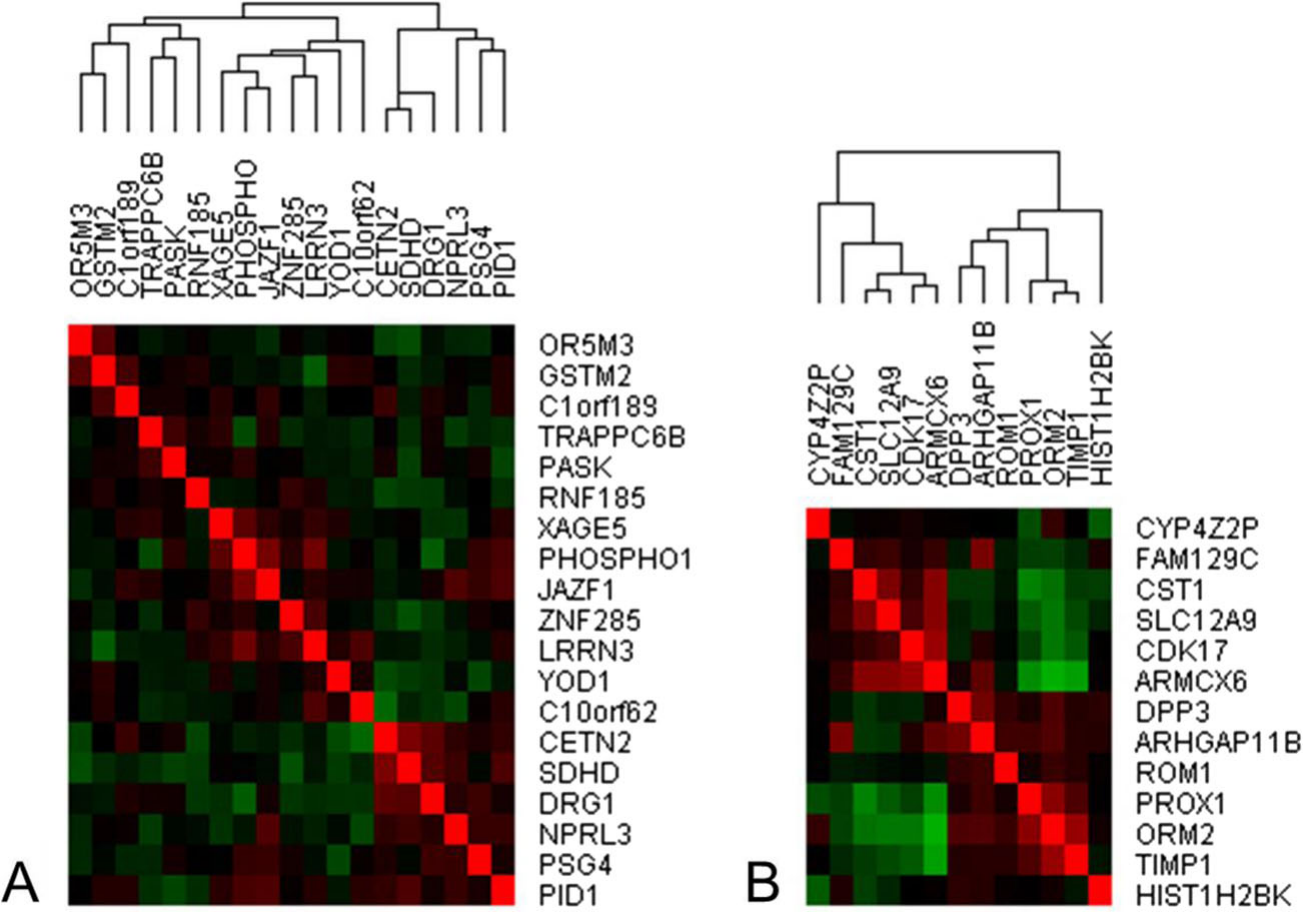
Prior transcriptional correlations between a subset of highly downregulated (**a**) and upregulated (**b**) genes in CNBP patients using 3900 human 2-colour microarrays. Using a matrix of transcriptional correlations derived from the analysis of 3900 human 2-colour microarrays from NCBI’s Gene Expression Omnibus (GEO), which includes data from a variety of control and experimental samples, gene–gene Pearson’s correlation coefficients were determined. In the 3900 microarrays used to perform gene–gene correlations, *PROX1*, *ORM2* and *TIMP1*, we found to positively correlate with each other, which was supported by our CNP data analysis. In the 2-colour microarray analysis, other upregulated genes, including *CST1*, *SLC12A9*, *CDK17*, *ARMCX6*, were usually negatively correlated (*green*) to *PROX1*, *ORM2* and *TIMP1* (the *brightest red squares* are the self–self comparisons along the diagonal). However, our analysis highlighted that both groups of genes were upregulated, thus providing evidence that *PROX1*, *ORM2* and *TIMP1*, which are highly correlated in previous experiments, may be associated with the pathophysiology of CNP and may function as CNP biomarkers. *ARHGAP11B* rho GTPase activating protein 11B, *ARMCX6* armadillo repeat containing X-linked 6; *C10orf62* chromosome 10 open reading frame 62; *C1orf189* chromosome 1 open reading frame 189; *CDK17* cyclin-dependent kinase 17; *CETN2* centrin, EF-hand protein, 2; *CST1* cystatin SN; *CYP4Z2P* cytochrome P450, family 4, subfamily Z, polypeptide 2, pseudogene; *DPP3* dipeptidyl-peptidase 3; *DRG1* developmentally regulated GTP binding protein 1; *FAM129C* family with Sequence Similarity 129, member C; *GSTM2* glutathione S-transferase mu 2 (muscle); *HIST1H2BK* histone cluster 1, H2bk; *JAZF1* juxtaposed with another zinc finger protein 1; *LRRN3* leucine rich repeat neuronal 3; *NPRL3* nitrogen permease regulator-like 3; *OR5M3* olfactory receptor, family 5, subfamily M, member 3; *ORM2* orosomucoid 2; *PASK* PAS domain containing serine/threonine kinase; *PHOSPHO1* phosphatase, orphan 1; *PROX1* prospero homeobox 1; *RNF185* ring finger protein 185; *PID1* phosphotyrosine interaction domain containing 1; *PSG4* pregnancy specific beta-1-glycoprotein 4; *ROM1* retinal outer segment membrane protein 1; *SDHD* succinate dehydrogenase complex, subunit D, integral membrane protein; *SLC12A9* solute carrier family 12 (potassium/chloride transporters), member 9; *TIMP1* tissue inhibitor of metalloproteinases 1; *TRAPPC6B* trafficking protein particle complex 6B; *XAGE5* X antigen family, member 5; *YOD1* YOD1 deubiquitinase; *ZNF285* zinc finger protein 285

We also analyzed prior correlations among differentially regulated genes from the 2-colour microarrays, with high up- and downregulation (Fig. 1). Within the most strongly upregulated genes, a subset of 3–*TIMP1*, *ORM2* and *PROX1* was highly correlated in other experiments. TIMP1 [28–32], ORM2 [28, 30] and PROX1 [33, 34] have all been found in proteomic studies in plasma, suggesting that they may have potential as circulating biomarkers. Literature-mining analysis of the three genes [35] identifies ANG-1 (angiopoietin, a regulator of postnatal angiogenesis) as their strongest commonality.

### Expression Analysis Refinement

*TIMP1*, which was highlighted by our correlation analysis, dipeptidyl peptidase 3 (*DPP3*) and melanocortin 1 receptor (*MC1R*), all exhibited a strong basis of literature supporting the role of these genes in pain pathways [36–39]. Similarly, both orosomucoid 2 (*ORM2*) and prospero homeobox 1 (*PROX1*) were present in the correlation analysis, though *PROX1* did not meet any of our other refinement criteria. Caspase 5 (*CASP5*) and NLR family CARD domain-containing protein 4 (*NLRC4*) were also upregulated in patients with CNBP, alongside other genes such as toll-like receptor 5 (*TLR5*) and clusterin (*CLU*) (Table 1). We also observed several downregulated transcripts in CNBP patients, including ring finger protein 185 (*RNF185*) and lin-28 homolog A (*LIN28A*).

### Plasma TIMP1 Quantification

**Fig. 2:**
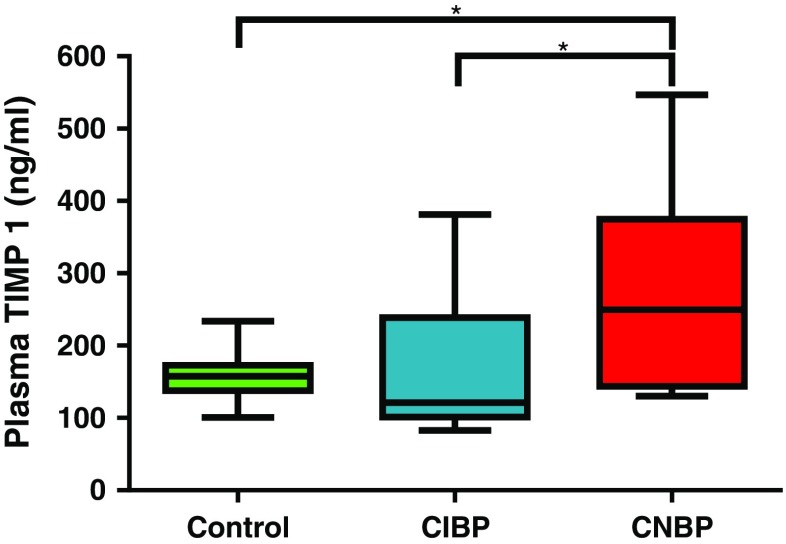
Plasma TIMP1 concentrations in healthy controls and patients with CIBP or CNBP. Analysis of plasma TIMP1 concentrations in healthy controls (*n = 10*), CIBP patients (*n = 12*) and CNBP patients (*n = 10*) was carried out using an ELISA. Diluted plasma samples were exposed to human TIMP1 monoclonal antibody coated wells and treated with human TIMP1 antibody conjugated to biotin. After Streptavidin–Peroxidase treatment, addition of substrate allows for colourmetric detection at 450 nm. Greater absorbance recordings correlate to higher plasma TIMP1 levels. *p = ≤0.05 (Mann–Whitney)

The mean (±SD) level of plasma TIMP1 in healthy control subjects was 157.3 (±33.2) ng/ml (range: 100.9–233.6 ng/ml); in contrast, the mean level in CNBP patients (±SD) was 278.4 (±131.4) ng/ml (range: 130.3–546.8 ng/ml). In patients with CIBP, the mean (±SD) was 147.8 (±75.55) ng/ml (range: 82.56–316.9 ng/ml). Plasma TIMP1 concentrations were therefore significantly elevated in patients with CNBP when compared to controls (*p* = 0.0433) and between patients with CNBP and CIBP (*p* = 0.0272) (Fig. 2). There was no significant change between controls and CIBP patients (*p* = 0.6682). When analysing controls, CNBP and CIBP patients together, significance of elevated TIMP1 was similarly observed (*p* = 0.0434). Plasma TIMP1 levels for controls and CNBP patients were moderately positively correlated to *TIMP1* mRNA levels isolated from whole blood (Pearson’s correlation, *R* = 0.68, *p* = ≤ 0.05). Age (*p* = 0.4980) and gender (*p* = 0.9948) covariates did not significantly influence TIMP1 levels, as determined by ANOVA and unpaired *t* test, repectively.

### SNL Dorsal Horn Expression Analysis

Genes found to be differentially regulated in human blood were subsequently analysed in the dorsal horn of Sprague–Dawley rats that had undergone L5 SNL, in conjunction with their sham counterparts (Table 2). *Timp1* expression was notably upregulated (*p* = 0.0058) 35 days post-SNL. This was accompanied by a strong trend for upregulation of *Mc1r* (*p* = 0.0847).

**Table 2:** qRT-PCR and ddPCR analysis of gene expression changes after SNL in Sprague–Dawley rats

Accession number	Gene name	Gene symbol	*p* value	FC in SNL
NM_053736	Caspase 4 ^a^	*Casp4*	0.1951	↑1.22
NM_053021	Clusterin	*Clu*	0.9990	1.00
NM_053748	Dipeptidyl-peptidase 3	*Dpp3*	0.4670	↓1.16
XM_006256260	Inducible T cell co-stimulator ligand	*Icoslg*	0.7920	↓1.16
NM_001109269	Lin-28 homolog A	*Lin28a*	0.7860	↑1.30
XM_006255795	Melanocortin 1 receptor^a^	*Mc1r*	0.0847	↑2.72
NM_001309432	NLR family CARD domain-containing protein 4^a^	*Nlrc4*	0.5242	↑1.15
NM_001168524	Rho GTPase activating protein 11A ^a^	*Arhgap11a*	0.8516	↓1.03
NM_001024271	Ring finger protein 185	*Rnf185*	0.3900	↓2.18
NM_053819	TIMP metalloproteinase inhibitor 1	*Timp1*	0.0058	↑2.19
NM_001145828	Toll-like receptor 5	*Tlr5*	0.6820	↑1.51

^a^Denotes analysis by ddPCR. The ortholog or closely related gene with high sequence similarity was selected as appropriate comparison to represent human gene. Genes not described here are either not present within the rat genome with no apparent ortholog or were not reliably detected for robust statistical analysis

## Discussion

In the search for biomarkers of pain in the blood, research has focused on proteomic analysis. It has been reported that the levels of serum biomarkers correlate with lower back pain and related functional impairment [40], and that the severity of polyneuropathy is associated with elevated tumor necrosis factor-alpha and interleukin-6 (IL-6) [41]. Increased IL-6 has also been postulated to be a predictive blood biomarker in herpes zoster, indicating propensity to develop post-herpetic neuralgia [42]. A similar study to this, using a CCI model of NP, found multiple changes in gene expression which correlated between blood and iLDQ, including the inducible T cell co-stimulator ligand (*Icoslg*) [19]. *ICOSLG* was upregulated in our CNBP cohort, but not in the SNL model of NP. This, however, may be explained by the use of a contrasting animal model of NP.

In order to further determine the viability of blood as a source of CLBP biomarkers, based on the results obtained, we initially established a list of genes differentially regulated in human blood in patients with CNBP. To refine and establish a list of candidate genes, we implemented an enrichment analysis including a combination of *p* value and fold change cutoffs. Although there are limitations to this approach, it did allow the identification of candidate genes for validation purposes that may have been eliminated with more stringent criteria. After analysis and refinement of these genes using our predetermined criteria, 10 of the 15 differentially regulated genes had existing associations with CNP, pain-related conditions or mechanisms underpinning pain. TIMP1, an inducible, soluble and secreted protein with cytokine-like properties [43], was significantly upregulated in the blood of CNBP patients, highlighted in our correlation analysis and was supported by a plethora of publications (Table 1). TIMPs function to inhibit the matrix metalloproteinases (MMPs), a group of zinc-dependent endopeptidases involved in extracellular matrix degradation, with several consequential roles in cell–cell interactions, migration and cell proliferation [44]. Such inhibition has been shown to reverse allodynia post-SNL [45], and after sciatic nerve injury, the MMP9/TIMP1 axis has been associated with the nerve regeneration process [46]. Cytokine-mediated changes in TIMP1 regulation have also been associated with permeability changes in the subendothelial basement membrane, facilitating neuro-immune interactions through leukocyte migration to the perivascular tissue [47]. Moreover, as a circulating prognostic marker, many studies have reported disease associations with the MMP9/TIMP1 axis. High serum MMP9 and low TIMP1 levels were associated with brain lesion formation in relapsing-remitting MS [48], and a decrease in the ratio was associated with interferon treatment [49]. This highlights the potential for circulating TIMP1 to reflect the activity of neurological pathology.

We also showed that *Timp1* is significantly upregulated in Sprague–Dawley ipsilateral dorsal horn, which supports its potential role in the mechanisms underpinning the maintenance or development of CNP and CNBP. Such upregulation has also been observed after rat spinal cord injury and in the dorsal root ganglion after sciatic nerve transection (SNT), 28 days after surgery, leading to suggestions that *Timp1* may be involved in pain persistence [36, 50]. Interestingly, this was observed in the absence of significantly upregulated MMP9, a target of TIMP1-mediated inhibition [51], which supports growing evidence that TIMPs may have MMP-independent functions [52, 53]. In addition, *Timp1* was found, alongside a cluster of secretion-related genes, to be upregulated in the spinal cord after CCI [37]. This upregulation was not observed in rats with complete Freund’s adjuvant induced inflammatory pain, which suggests that *Timp1* expression may be discriminatory neuropathic and inflammatory pain, thereby lending further support to our determination that TIMP1 was significantly higher in the plasma of CNBP patients, than those with CIBP.

*DPP3*, an enkephalinase and single member of the M49 family of metallopeptidases, exhibits a strong association with the mechanisms that underpin nociception and was also notably upregulated in patients with CNBP. DPP3 plays a critical role in the degradation of enkephalin within the pain modulatory system [38] and has been highlighted as a potential target for the pharmacological management of pain [54], with inhibition of DPP3 by the endogenous opioid peptide spinorphin demonstrating analgesia in mice [55]. Interestingly, DPP3 activity in human cerebrospinal fluid was reduced in subjects with acute pain when compared to pain free subjects [56]. The structural conformations of DPP3 and the subsequent changes upon substrate binding have now been elucidated, giving impetus for furthering the development of novel DPP3 inhibitors [38, 57]. The potential of DPP3 as a pharmacological target, and the potential role of DPP3 as a biomarker for CNBP, is therefore highly supportive and certainly warrants further investigation.

Our results also show an upregulation of *CASP5* and *NLRC4*. Caspases are endoproteases and regulators of cell death and inflammation and undergo activation following the detection of highly conserved pathogen-associated molecular patterns by toll-like receptors (TLRs) and other pattern-recognition receptors, such as danger-associated molecular patterns (DAMPs). DAMPs recognise a variety of stimuli, including ATP-mediated P2X7 receptor activation, amyloid-β and monosodium urate, leading to associations with AD [58, 59] and gout-associated sterile inflammation [60]. This highlights the diverse range of triggers for the formation of the inflammasome, a multiprotein complex and component of the innate immune system [61]. In addition, caspase-5 was shown to be upregulated in peripheral blood of fibromyalgia patients reporting high pain, in contrast to those with low pain [62] and in patients with ankylosing spondylitis [63].

It has also been shown that mice deficient in NLRC4 inflammasomes showed attenuated carrageenan induced mechanical and thermal acute inflammatory hyperalgesia which coincided with reduced levels of interleukin-1β (p17) and caspase-1 [64]. Moreover, after rat cervical spinal cord injury, CASP11 (CASP4), the ortholog of human CASP5, was upregulated and activation of the NALP1 inflammasome, which incorporates CASP11, was observed. Antibody-mediated neutralisation of a component of this multiprotein complex, ASC, also resulted in notable tissue sparing and functional improvement [65]. Taken together with our expression analysis, these findings suggest that upregulation of *CASP5* and *NLCR4* may be useful indicators of injury and inflammatory processes, but further clarification is required to determine their specificity to CNBP.

*MC1R* was significantly upregulated in patients with CNBP and trended strongly towards upregulation in the SNL model of NP. The endogenous melanocortin receptor agonist, alpha melanocyte-stimulating hormone (α-MSH), is derived from post-translational processing of proopiomelanocortin. Plasma levels of α-MSH are tightly regulated, but increases have been observed in inflammatory disorders, at localised regions of inflammation [66] and after central administration of α-MSH, reduced peripheral inflammation has been observed [67]. In addition, α-MSH has also been shown to inhibit nitric oxide production in a murine macrophage cell line after lipopolysaccharide and interferon-gamma (IFN-γ) stimulation and exhibited autocrine function leading to modulation of the inflammatory response [68].

Point mutations in *MC1R* are often cited as responsible for the red-haired phenotype [69]. Individuals with this phenotype have been shown to exhibit greater anesthetic requirement [70] and increased sensitivity to thermal pain [71]. It has also been determined that females with two variant *MC1R* alleles experience enhanced analgesia with pentazocine, a kappa-opioid receptor agonist [72]. Antagonism of the melanocortin system has been associated with a reduction in mechanical and cold allodynia and the reversal of morphine-induced hyperalgesia [73–75]. Conversely, a role for MC1R in sex-specific variation in inflammatory pain but not CNP has also been shown [76]. The influence of MC1R and the wider melanocortin system on pain perception is clearly diverse, with multiple studies highlighting varying associations between the melanocortin system, pain and sex-specificity. Further mechanistic and biomarker analysis must be undertaken to elucidate and validate the role of MC1R, which showed upregulation in both CNBP patients and the SNL model of NP.

We also observed differential regulation of a number of other genes in the blood of patients with CNBP. After SNT, activation of the complement cascade has been observed alongside increased dorsal horn *Clu* mRNA expression [77]. We did not, however, observe a similar upregulation of *Clu* in the SNL model of NP. Upregulation of toll-like receptor 5 (*TLR5*) was also observed in CNBP patients. Recent studies have highlighted a potential role for TLR5 in NP, with *tlr5* ^−/−^ mice exhibiting reduced tactile allodynia after L5 SNL [78]. Although CLU and TLR5 are examples of differentially regulated genes in human blood that did not translation to the SNL animal model, with such variability in the animal pain models available [17], further analysis using alternative models of NP would be required to assess these genes as potential translational biomarkers.

We have therefore identified a range of genes differentially regulated in the blood of patients with CNBP, and of these, *TIMP1*, *DPP3* and *MC1R* possess a relatively strong literature basis supporting their role in CNP. Further scrutiny of these genes has facilitated the development of the list of candidate genes that warrants further investigation. The presence of differential regulation of *Timp1* and *Mc1r*, in the rat dorsal horn following L5 SNL, suggests that they may function as translational biomarkers and may be perturbed in the mechanisms underpinning CNP in the dorsal horn. Although there are limitations to the use of dorsal horn tissue as opposed to the dorsal root ganglion, however the dorsal horn does provide a disease relevant tissue that has many potential targets for the development of novel analgesics [22]. Moreover, many drug targets that have passed preclinical tests fail in human trials; this is partly due to the lack of robust translation from the human disease to animal model; here, we show potential biomarkers of CNP in humans to be present in a rat model of CNP and could function as indicators in drug and biomarker development. We therefore present TIMP1 as potential translational biomarker which is able to differentiate between patients with CNBP and CIBP. Future work and further analysis are now required to validate these findings with the aim of deciphering a molecular signature which, alongside traditional diagnostic methods, has the ability to vastly improve the diagnosis of CNP subtypes. This will include to explore the role of these biomarkers in other CNP subtypes, as well as expanding the investigation into preclinical systems, including different tissues and animal model of neuropathic pain.

## Electronic supplementary material

Supplementary table 1 (XLSX 15 kb)

Supplementary table 2 (DOCX 17 kb)

Supplementary table 3 (XLSX 42 kb)
