# Supplementary material for: The Development of Translational Biomarkers as a Tool for Improving the Understanding, Diagnosis and Treatment of Chronic Neuropathic Pain
Source: Mol Neurobiol. 2017 Mar 30;55(3):2420–30. doi: 10.1007/s12035-017-0492-8 (PMC5840239; doi:10.1007/s12035-017-0492-8)
Supplement: Supplementary file 2 — (DOCX 17 kb) [file 12035_2017_492_MOESM2_ESM.docx]

**Supplementary Table 2 – Primer sequences for gene expression analysis in the SNL model of neuropathic pain ^a^**

| Gene Symbol | Accession Number | Primer | Sequence |
| --- | --- | --- | --- |
| *Arhgap11a* | NM_001168524 | Forward Primer | 5’ TGTTGCTGTCGTGCCTTATG 3’ |
|  |  | Reverse Primer | 5’ CAAGATTGCTGCTGTCCATTT 3’ |
| *Clu* | NM_053021 | Forward Primer | 5’ GGGCGATGACCAGTACCTT 3’ |
|  |  | Reverse Primer | 5’ TCAAACAGCTTCACCACCAC 3’ |
| *Dpp3* | NM_053748 | Forward Primer | 5’ GATCCGGTCAGTGGGCAAA 3’ |
|  |  | Reverse Primer | 5’ CCTGCAACCACATCCCCT 3’ |
| *Elf3* | NM_001024768 | Forward Primer | 5’ TCTTCGTTCAGAGGCTGTGG 3’ |
|  |  | Reverse Primer | 5’ CGTTCCAGGATCTCCCGTTT 3’ |
| *Icoslg* | XM_006256260 | Forward Primer | 5’ TCAGTACGCCTGTCATCAGC 3’ |
|  |  | Reverse Primer | 5’ ACGTCATACAGGCCCAACTC 3’ |
| *Lin28a* | NM_001109269 | Forward Primer | 5’ GTATTGGGAGTGAGCGACGG 3’ |
|  |  | Reverse Primer | 5’ ACAGTTGTAGCACCTGTCTCC 3’ |
| *Rnf185* | NM_001024271 | Forward Primer | 5’ TGGCTTTCAGATGTCTTTTGGA 3’ |
|  |  | Reverse Primer | 5’ CAAACAGGAAGAGGCGTGAC 3’ |
| *Timp1* | NM_053819 | Forward Primer | 5’ CCTGGCATAATCTGAGCCCT 3’ |
|  |  | Reverse Primer | 5’ TTTGCAAGGGATGGCTGAAC 3’ |
| *Tlr5* | NM_001145828 | Forward Primer (Ann: 62.4^o^C) | 5’ CTGTCTGACCTCAAGCGTGT 3’ |
|  |  | Reverse Primer (Ann: 62.4^o^C) | 5’ GGGCCACCTCAAATACTGCT 3’ |

**^a^** Sequences were not available for *Casp4*, *Mc1r*, *Nlrc4*, *Orm1* (BioRad PrimePCR Assays for ddPCR) and for *Atp5b* and *Ubc* (Primer Design geNORM Assays)
